# Horizontal Ridge Augmentation With Symphyseal Autograft: Clinical and Radiographic Analysis

**DOI:** 10.7759/cureus.97153

**Published:** 2025-11-18

**Authors:** Sangeeta Rai, Ashutosh Dixit, Vasudha Gupta, Shambhavi Kumar

**Affiliations:** 1 Dentistry, All India Institute of Medical Sciences, Rishikesh, Rishikesh, IND; 2 Dentistry, All India Institute of Medical Science, Rishikesh, Rishikesh, IND

**Keywords:** autogenous grafts, block graft, dental implants, horizontal ridge augmentation, mandibular symphysis

## Abstract

Horizontal ridge augmentation with symphyseal autograft is a reliable method of treating alveolar ridge deficiencies in the anterior mandible. Autogenous bone grafts from intraoral donor sites have been regarded as the gold standard for reconstructing jaws. The symphysis is an easily available source of corticocancellous bone with good osteogenic, osteoinductive, and osteoconductive characteristics. This case report illustrates the horizontal ridge augmentation with symphyseal block grafts successfully before implant placement. Fixation screws were used to fix harvested blocks to the ridged defect, along with particulate bone grafts and barrier membranes to provide stability and prevent resorption. This technique provided outstanding horizontal bone gain with a good foundation for placing the implant in otherwise compromised locations. Case selection, accurate surgical technique, and respect for biologic principles are paramount to achieving the best results. On balance, symphyseal autografts are still a solid choice for horizontal ridge augmentation in implant dentistry.

## Introduction

Horizontal ridge augmentation is a foundation in implant dentistry, treating the frequent problem of alveolar bone loss that usually results from tooth extraction, periodontitis, or trauma. Without sufficient alveolar width, it becomes much more problematic to obtain optimal implant stability and esthetics [1]. Of grafting choices available, e.g., guided bone regeneration (GBR), distraction osteogenesis, and alloplastic or xenogenic material substitutes-autogenous bone grafts are globally accepted as the gold standard because of their intrinsic osteogenic, osteoconductive, and osteoinductive properties [1,2]. 

Intraoral donor sites, most notably the mandibular symphysis, provide unique advantages for autograft harvesting: easy surgical access, short operative time, low morbidity, and bone with good membranous characteristics to aid in revascularization and less resorption than with extraoral sources [2,3]. Symphyseal autografts are thus particularly well-suited to the augmentation of horizontal alveolar deficiencies of the aesthetic anterior regions.

Numerous clinical reports have evidenced horizontal bone gain of about 3 to 5 mm with symphyseal block graft. For instance, a case series of severe maxillary anterior ridge defects had a mean gain of 3.9 mm and uneventful implant placement after augmentation [4]. Systematic reviews also confirm that horizontal augmentation procedures with autogenous block grafts-either alone or in combination with particulate bone and membranes-are highly predictable [5]. Recent developments are computer-assisted harvesting and positioning of symphyseal cortical *shells*, which rationalize placement precision and improve surgical security, even if the bone gain is statistically equivalent to that of traditional methods [6]. Another breakthrough is the L-shaped symphyseal bone block, specifically designed for 3D augmentation (horizontal and vertical). In a recent prospective investigation, the method gained an immediate horizontal approximately 4.17 ± 0.77 mm (with a slight decrease to 3.52 ± 0.75 mm after six months) and a vertical gain of up to ~6.5 mm - again evincing its potential in complicated atrophic ridges [2]. The selection of the grafting method, whether to do staged or simultaneous implant placement, is still important. Staged techniques are more likely to provide more vigorous and predictable results. Implant survival rates, as reported, vary between 93.5% and 100%, with horizontal gains ranging from 3.4 to 5.0 mm in augmented ridges [5]. Surgical principles like flap design, optimal fixation, and tension-free closure are important in avoiding complications such as graft exposure, membrane collapse, or soft tissue dehiscence [7]. In conclusion, horizontal ridge augmentation with symphyseal autografts is still a very successful modality of implant-based rehabilitation. With consistent bone gain, predictable implant results, and progressive techniques that improve accuracy and increase indications, symphyseal block grafting is still a favored approach to ridge deficiencies - particularly long-term esthetic regions.

## Case presentation

This case report presents localized anterior ridge augmentation using an autogenous block graft from the mandibular symphysis to restore ridge dimensions before implant placement.

**Figure 1:**
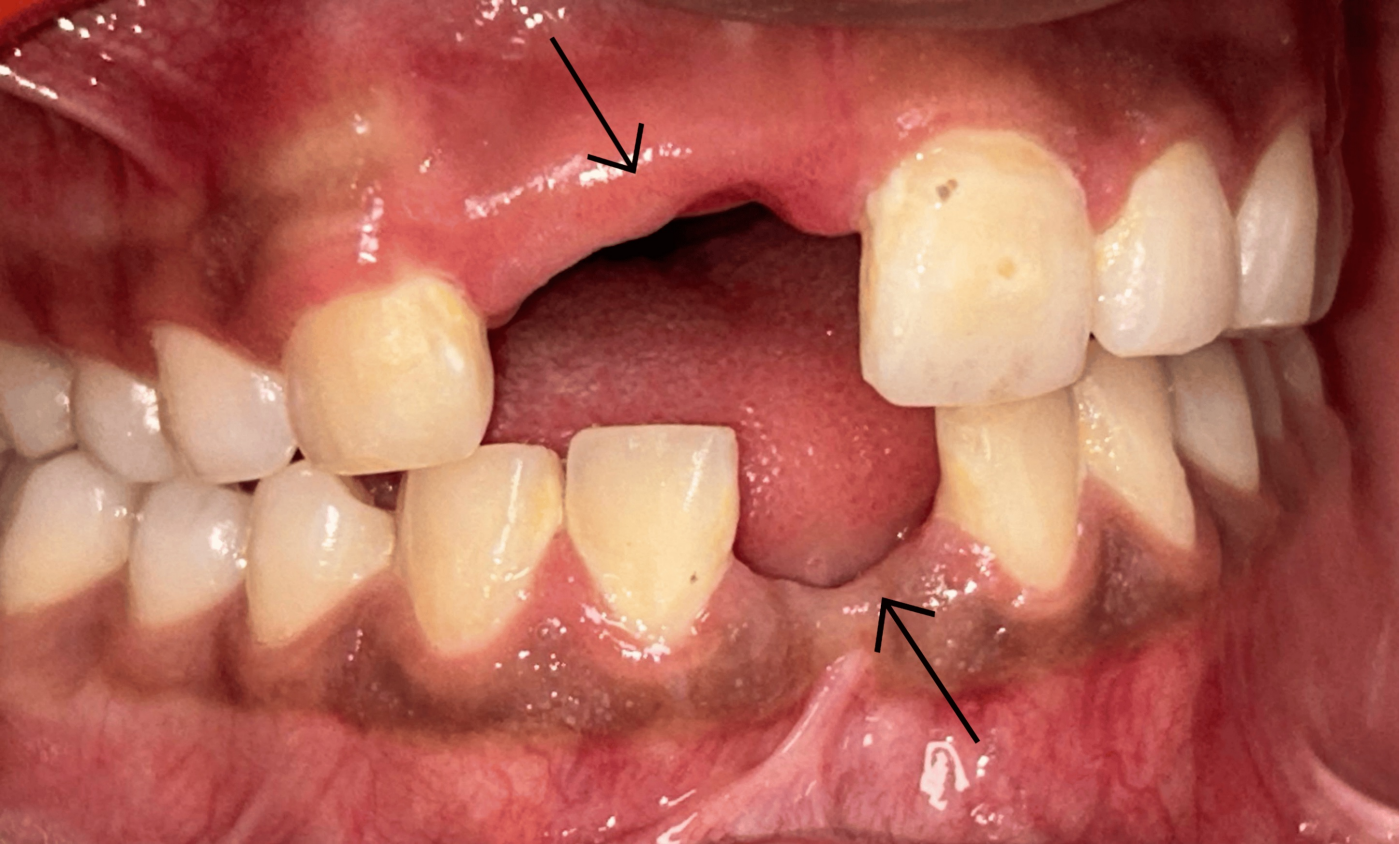
Preoperative clinical view.

A 19-year-old adolescent patient reported a desire for fixed replacement of missing teeth. He had a history of trauma five years ago, resulting in the loss of maxillary and mandibular anterior teeth, and has been wearing a removable prosthesis since then. The patient was in good systemic health and did not report any deleterious or parafunctional habits. Clinical examination revealed a marked reduction in crestal width at the edentulous site, compromising the ability to place an implant in an ideal prosthetic position (Figure 1).

**Figure 2:**
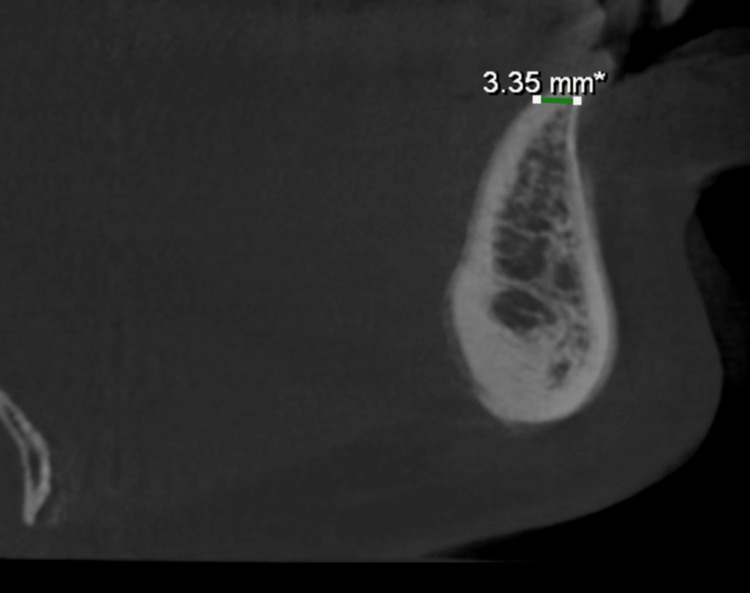
CBCT (sagittal view) showing a preoperative mandibular ridge width of 3.35 mm. CBCT, cone-beam computed tomography

**Figure 3:**
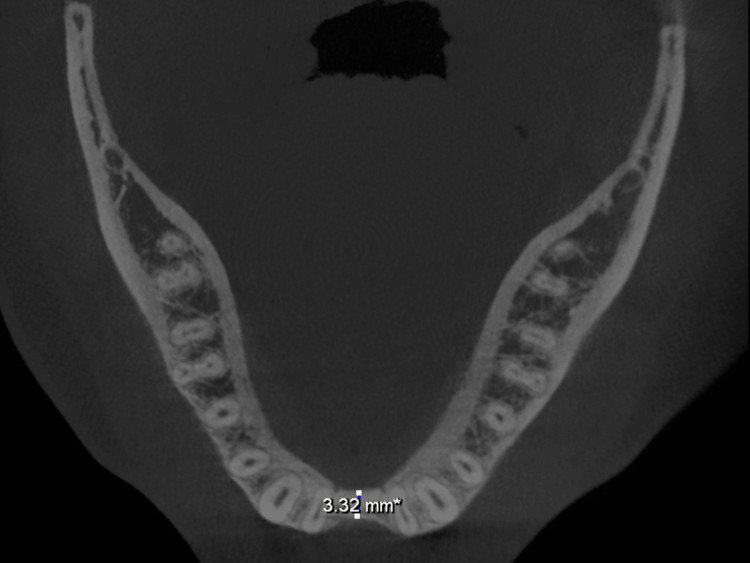
CBCT (axial view) indicating a preoperative mandibular ridge width of 3.32 mm. CBCT, cone-beam computed tomography

**Figure 4:**
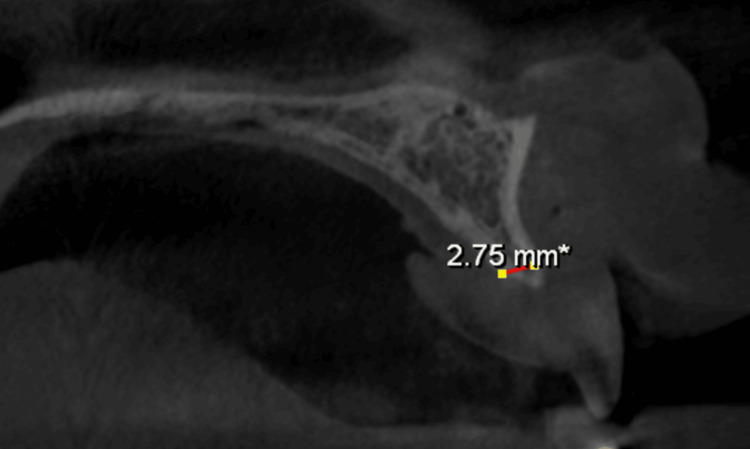
CBCT (sagittal view) indicating a preoperative maxillary ridge width of 2.75 mm. CBCT, cone-beam computed tomography

**Figure 5:**
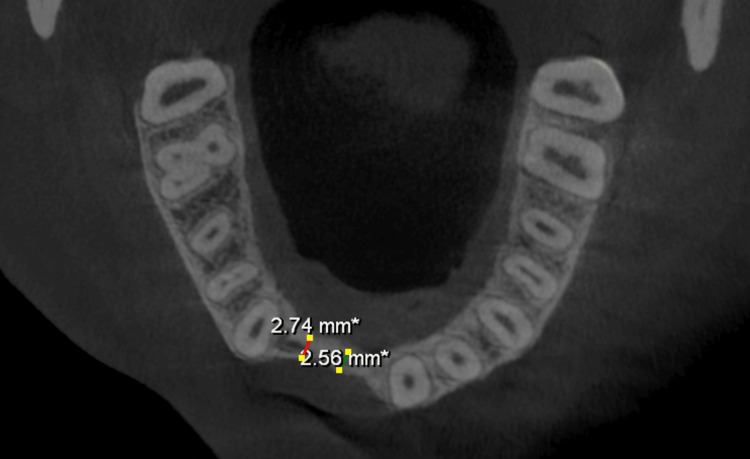
CBCT (axial view) indicating a preoperative maxillary ridge width of 2.56 mm. CBCT, cone-beam computed tomography

Preoperative assessment included standard blood tests, such as complete blood counts, viral markers for HIV, hepatitis B, and hepatitis C, coagulation tests, and blood glucose levels. Radiographic examination was performed with cone-beam computed tomography (CBCT) and orthopantomography (OPG). CBCT imaging verified gross horizontal ridge deficiency. CBCT measurements of the edentulous regions indicated a preoperative ridge width of about 2.56-3.35 mm in both maxilla and mandible (Figures 2, 3, 4, 5).

**Figure 6:**
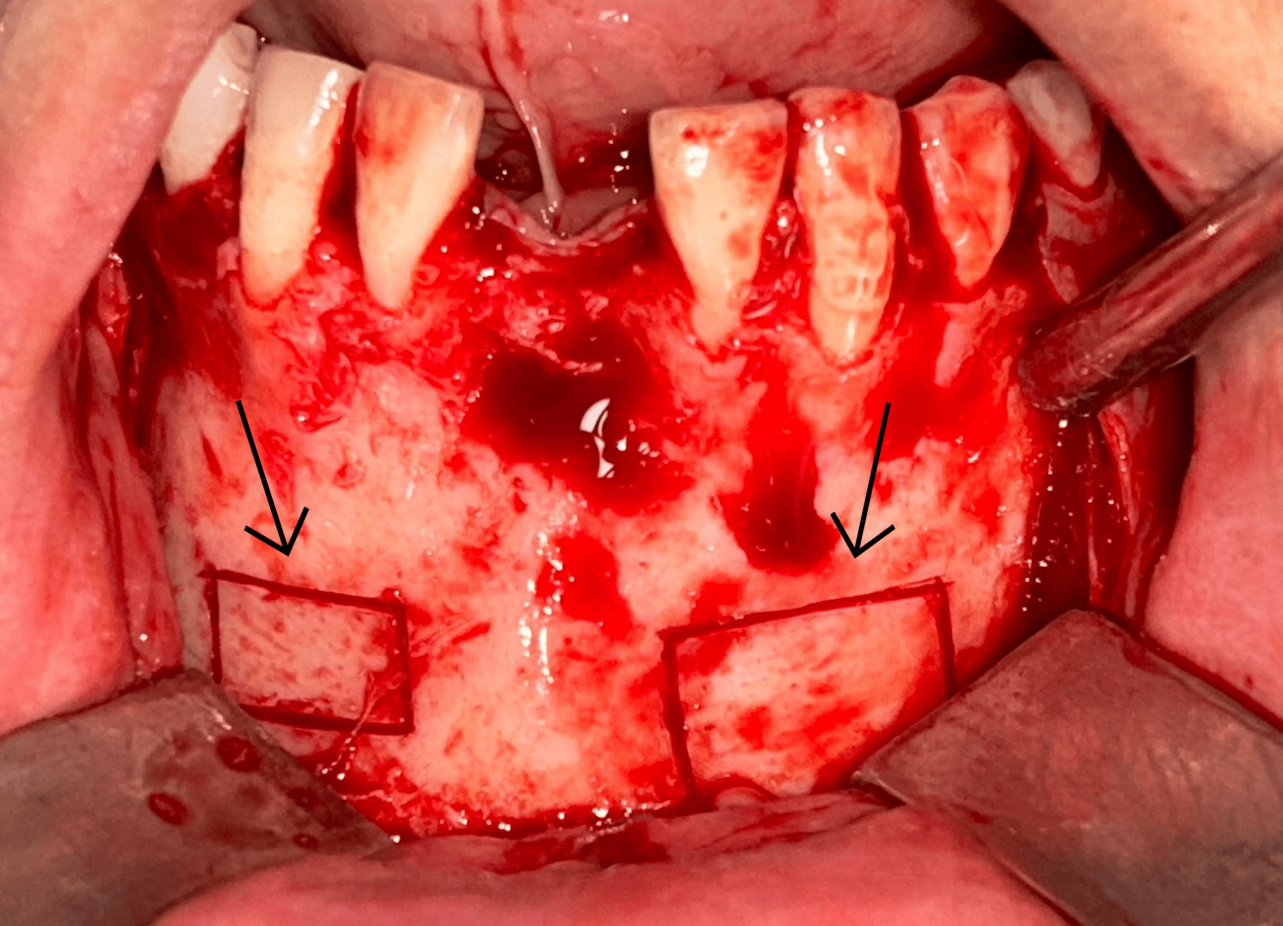
Block graft harvesting using a piezoelectric device.

The treatment included the harvesting of an autogenous cortical block graft from the mandibular symphysis under local anesthesia (2% lignocaine with 1:100,000 epinephrine). The graft was harvested as per Misch's rule of 5's with a piezoelectric device (Acteon, England). Two 10 x 15 mm and 8 x 10 mm rectangular monocortical plates were harvested (Figure 6).

**Figure 7:**
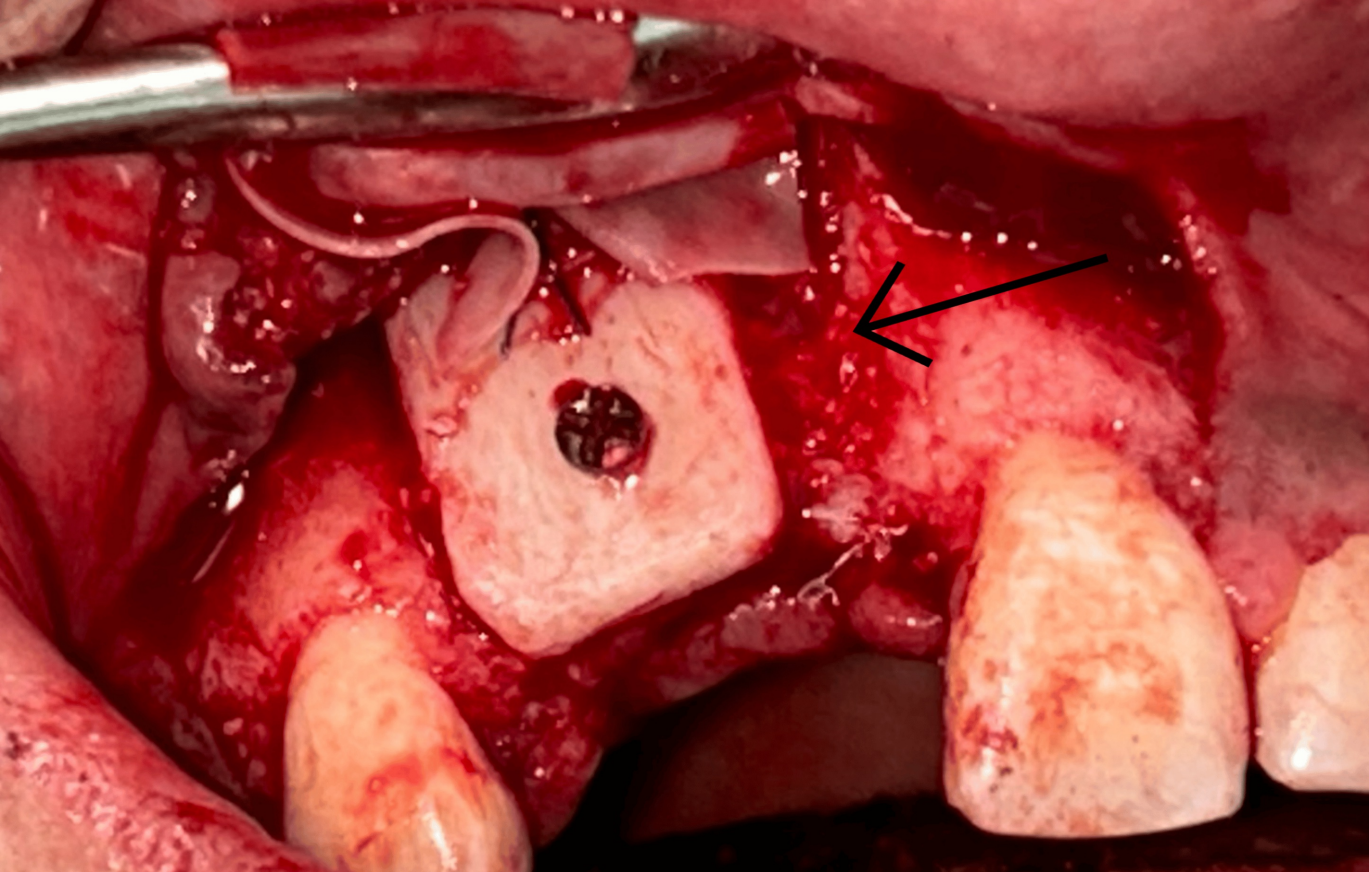
Block graft fixation with an osteosynthetic screw on the maxillary ridge.

**Figure 8:**
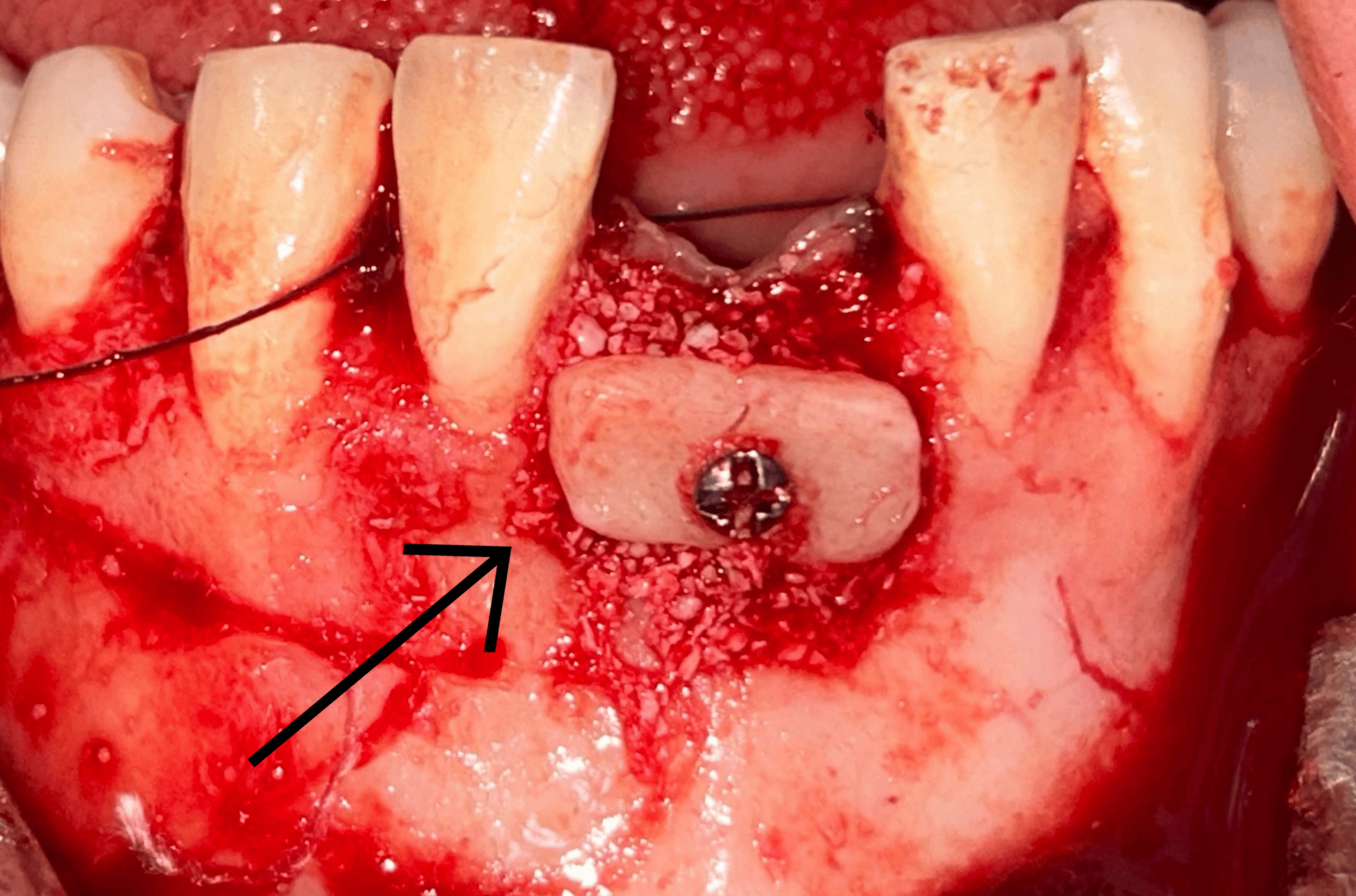
Block graft fixation with an osteosynthetic screw on the mandibular ridge.

**Figure 9:**
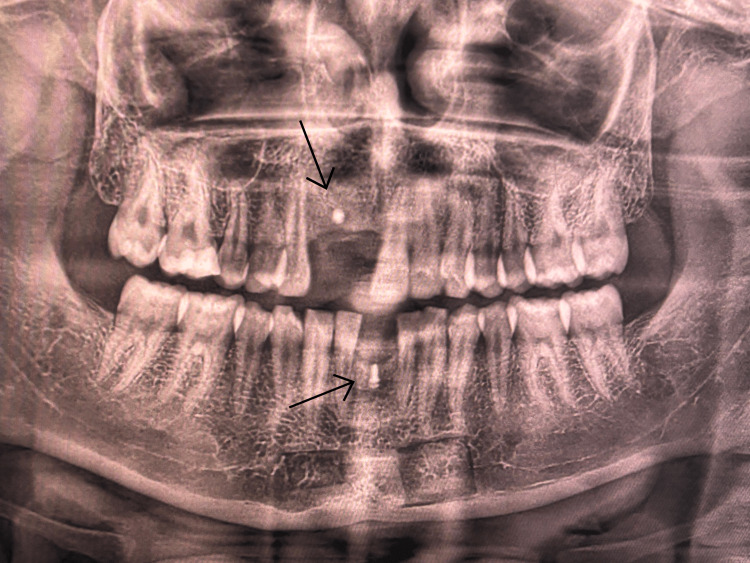
Immediate postoperative orthopantomogram (OPG).

A midcrestal incision at the recipient site, including intrasulcular extensions, with one tooth mesial and distal to the defect region. A full-thickness flap was reflected subsequently to reveal the recipient site. Decortication of labial cortical bone at the recipient site was carried out in order to improve revascularization. The harvested bone blocks were accurately shaped to conform to the defect and stabilized onto the residual ridge with a single titanium fixation screw that was 1.5 mm in diameter (Figures 7-8). To remove voids and facilitate graft integration, a particulate bovine-derived xenograft (Fix Oss, Synerheal Pharmaceuticals, Chennai, India) was seated about the block and along the ridge. A resorbable collagen membrane (HEALIGUIDE-Advanced Biotech Products, Chennai) was positioned over the graft. To allow tension-free closure, the buccal flap was released through periosteal scoring, and the surgical area was closed using a combination of horizontal mattress and single interrupted sutures (Ethicon Mersilk, Ethicon Inc., Raritan, NJ). Immediate postoperative OPG was taken (Figure 9). 

Routine postoperative care with antibiotics (500 mg amoxicillin + 125 mg clavulanic acid combination), anti-inflammatory drugs (400 mg ibuprofen), and chlorhexidine mouth rinses (0.2%) was administered. Preoperative and six-month postoperative measurements of bone width at the time of implant placement were taken using CBCT scans.

**Figure 10:**
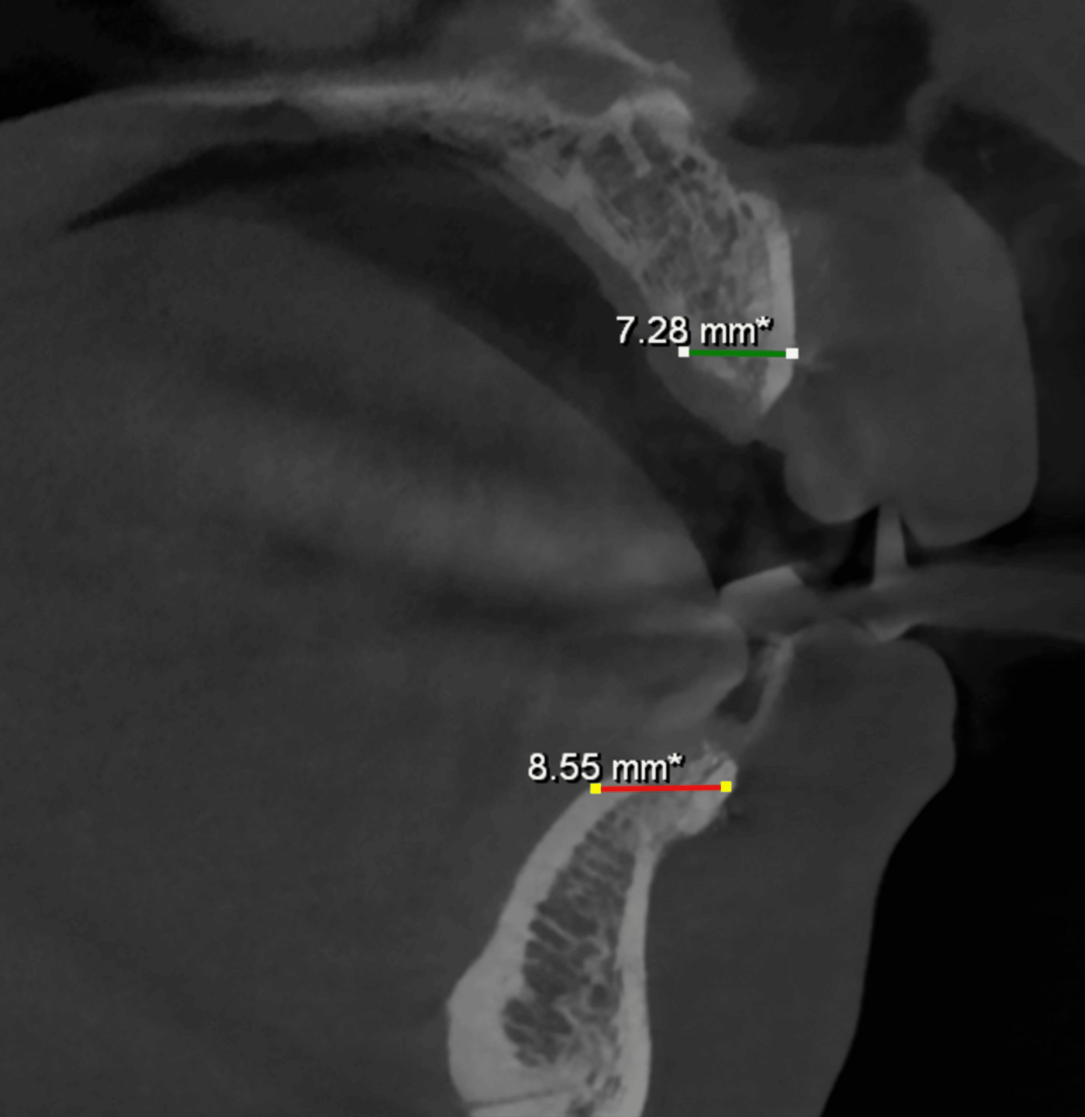
Postoperative CBCT (six months later) showing sagittal views of the maxillary and mandibular ridge widths, measuring 7.28 and 8.55 mm, respectively. CBCT, cone-beam computed tomography

**Figure 11:**
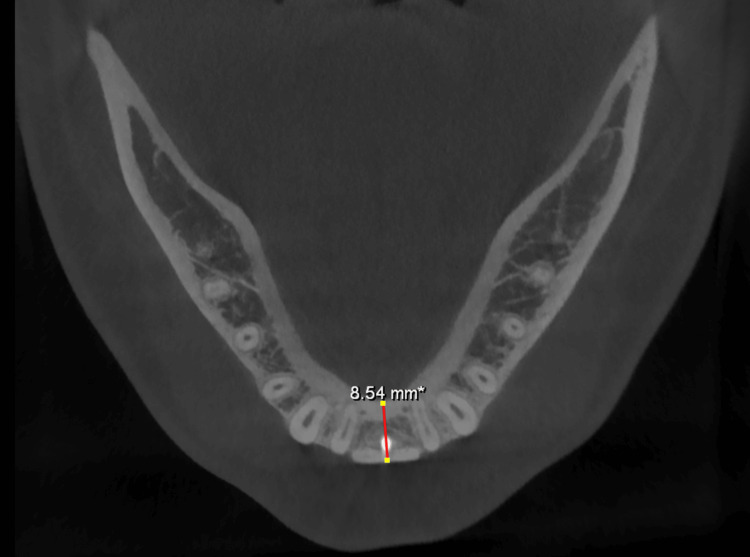
Postoperative CBCT (six months later) - axial view showing a mandibular ridge width of 8.54 mm. CBCT, cone-beam computed tomography

**Figure 12:**
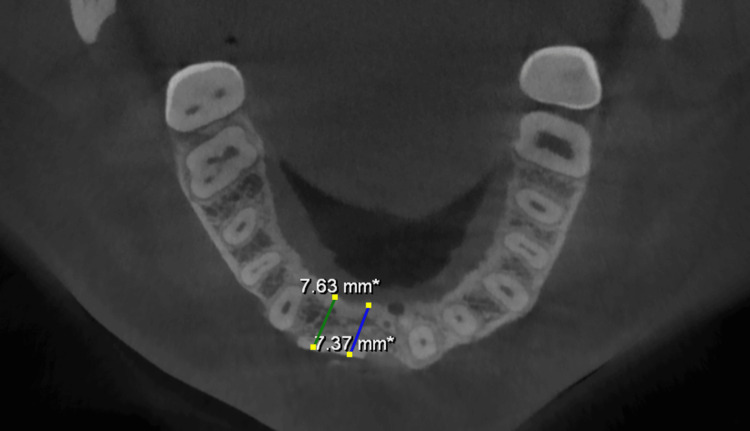
Postoperative CBCT (six months later) - axial view showing a maxillary ridge width of 7.63 mm. CBCT, cone-beam computed tomography

All the sites augmented at six months had adequate ridge width to allow appropriate implant placement in the prosthetically guided position (Figures 10, 11, 12). There was no graft exposure, soft tissue dehiscence, or other complications at any time during healing. Implants were inserted with appropriate primary stability.

**Figure 13:**
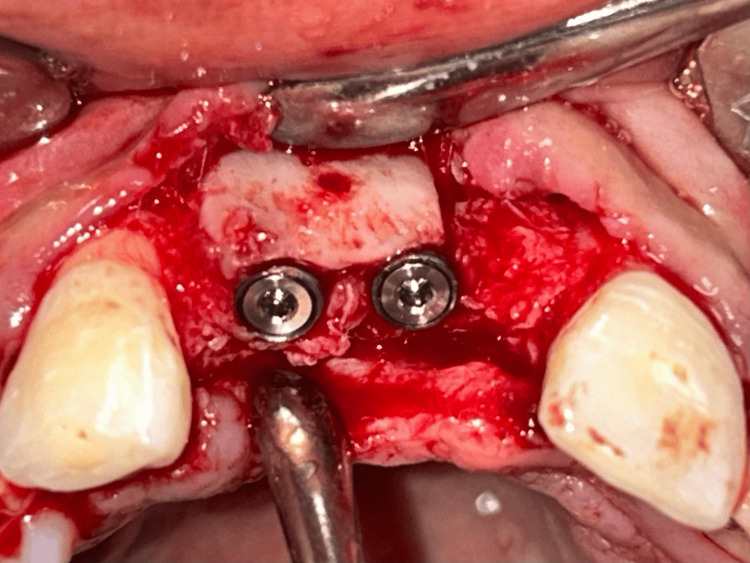
Implant placement in the maxillary region.

**Figure 14:**
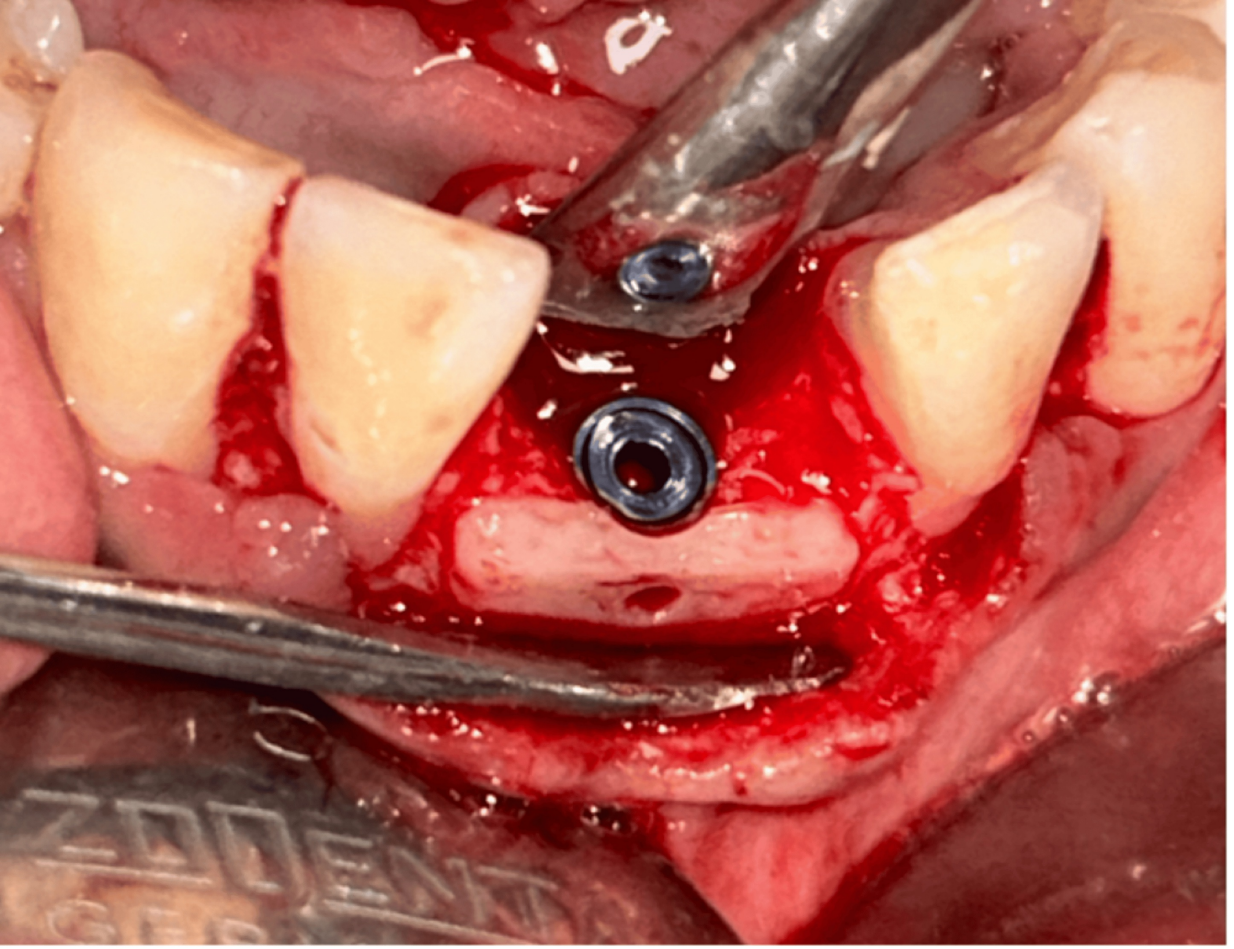
Implant placement in the mandibular region.

**Figure 15:**
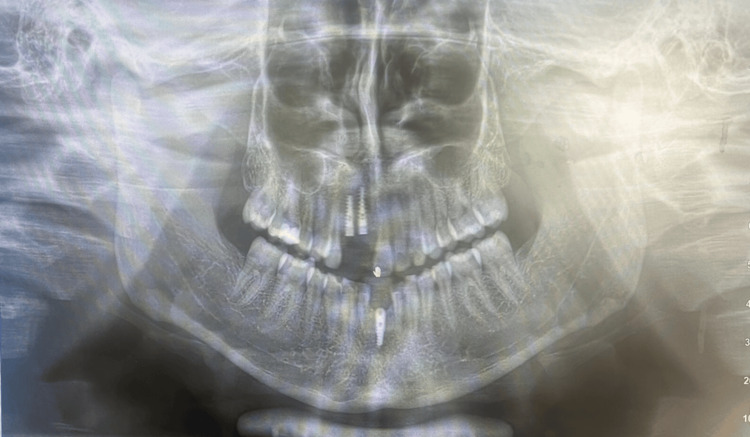
Post-implant placement orthopantomogram (OPG).

Implants (Bioline, Berlin, Germany) of sizes 3.75 x 11.5 mm and 3.3 x 11.5 mm were inserted in the region of maxillary 11 and 12 teeth, respectively (Figure 13). An implant (Bioline, Berlin) of size 3.3 x 11.5 mm was inserted in the region of mandibular 31 (Figure 14). OPG was taken after the implant placement (Figure 15). 

## Discussion

In this case report, a staged procedure was followed for anterior maxillary alveolar ridge augmentation. In the first surgical stage, block bone grafts were taken from the mandibular symphysis and fixed in the defect area. Six months of healing were allowed after that. Dental implants were placed. The procedure was highly successful in both esthetic and comfort for the patient and resulted in a mean buccolingual ridge width gain of about 3-4 mm.

Horizontal ridge augmentation with mandibular symphysis autografts is still a proven method in implant dentistry, providing tremendous clinical advantage and predictable results. As compared to GBR and other grafting materials, autogenous bone continues to be the gold standard because it possesses intrinsic osteogenic, osteoinductive, and osteoconductive properties [8]. Clinically, symphyseal grafts allow for significant horizontal bone gain, as supported by comparative research. One study reported a mean buccolingual increase in ridge height of 2.49 mm using symphysis grafts, outperforming ramus grafts by a significant margin at 1.48 mm [9]. Long-term implant survival in augmented sites horizontally with the use of block grafts has been demonstrated to occur between 96.9% and 100%, reflecting the efficacy of the technique [10]. Nevertheless, these benefits need to be properly weighed against donor site morbidity.

Symphysis harvesting carries a greater risk for an increased incidence of temporary and permanent sensory disturbance affecting the lower lip, chin skin, mucosa, and even the vitality of anterior teeth [11,12]. Sensory deficit was reported in 40.5% of patients by one study, with 13.5% having permanent disturbances; this is considerably higher than that seen for ramus donor sites [11]. Also, aesthetic complications like changes in the contour of the chin have occurred in up to one-third of cases in some reports [11]. In spite of these donor-site complications, harvesting from the symphysis offers practical benefits such as ease of access, good quality corticocancellous bone, and excellent osteogenic potential, thus making it well-adapted for anterior ridge defects [13]. Minimal graft resorption and smooth healing occurred in an early study, allowing successful placement of an implant following four months [13].

The harvesting process involves keeping the apices of mandibular incisors, mental foramen, and the inferior mandibular border at a safe distance to prevent structural and nervous injury. Misch et al. described a scientific and safe method for harvesting a bone block graft from the mandibular symphysis that would avoid damage to essential neurovascular structures of the area [13]. Based on his recommendations, the superior osteotomy must be set about 5 mm below the apices of the anterior teeth in order not to interfere with the tooth roots and the mandibular incisive canal. The inferior osteotomy must be at least 5 mm above the lower border of the mandible, and the vertical cuts must be at least 5 mm away from either side of the mental foramen. The depth of osteotomy must be from the outside cortical plate to the inner cortical layer to produce a monocortical graft safely. This is called the *rule of 5's *[14]. Misch et al. [13] said the safety guidelines recommended cannot always be followed for all patients due to insufficient bone height in the area of the symphysis.

Actually, compliance with these parameters was reported to put the mandibular incisive canal at risk in about 57% of cases. Pommer et al. [15] presented new safety guidelines for bone graft harvesting from the chin area to reduce the risk of injury to the mandibular incisive canal. By following these new parameters, damage risk can be lessened to about 16%. The suggestions are to have a graft depth of 4 mm, at least 8 mm distance from the anterior teeth apices, and keep more than 5 mm of bone above the lower border, and not approach the mental foramen [14]. Symphyseal grafts undergo some degree of resorption; however, this is less pronounced than in iliac crest grafts because they are of membranous origin. The retention of grafts using fixation screws, the application of barrier membranes, and achieving adequate tension-free closure all contribute to improved graft stability and integration.

## Conclusions

Rehabilitation of atrophic maxillary and mandibular ridges in this case report was complicated by marked bone loss. Ridge augmentation was obtained by taking an autogenous bone graft from the mandibular symphysis, which gave a good basis for subsequent implant placement. The operation resulted in a considerable increase in ridge width, enabling ideal implant stability. Autogenous bone grafts are still considered the standard against which all other ridge augmenting materials are compared, due to their enhanced biological properties and yet very predictable results.
